# Timing of Elective Cholecystectomy After Acute Cholecystitis: A Population-based Register Study

**DOI:** 10.1007/s00268-022-06772-x

**Published:** 2022-10-24

**Authors:** Agnieszka Popowicz, Lars Enochsson, Gabriel Sandblom

**Affiliations:** 1 Department of Clinical Sciences, Intervention and Technology (CLINTEC), Trauma and Emergency Surgery, Karolinska Institute, Karolinska University Hospital, SE-141 52 Solna, Stockholm Sweden; 2 Department of Surgical and Perioperative Sciences, Surgery, Umeå University, Umeå, Sweden; 3 Department of Clinical Science and Education, Department of Surgery, Södersjukhuset, Karolinska Institute, Stockholm, Solna, Stockholm Sweden

## Abstract

**Background:**

Acute cholecystectomy is standard treatment for acute cholecystitis. However, many patients are still treated conservatively and undergo delayed elective surgery. The aim of this study was to determine the ideal time to perform an elective cholecystectomy after acute cholecystitis.

**Methods:**

All patients treated for acute cholecystitis in Sweden between 2006 and 2013 were identified through the Swedish Patient Register. This cohort was cross-linked with the Swedish Register for Gallstone Surgery, GallRiks, where information on surgical outcome was retrieved. The impact of the time interval after discharge from hospital to elective surgery was analysed by multivariate logistic regression adjusting for gender and age.

**Results:**

After exclusion of patients not subjected to surgery, not registered in GallRiks and patients treated with acute cholecystectomy, 8532 remained. This cohort was divided into six-time categories. Using the first time interval < 11 days from discharge to elective surgery as the reference category the chance of completing surgery with a minimally invasive technique was increased for all categories (*p* < 0.05). The risk for perioperative complication and cystic duct leakage was reduced if surgery was undertaken > 30 days after discharge (both *p* < 0.05). The risk for bile duct injury was significantly increased if the procedure was undertaken > 365 days after discharge (*p* = 0.030). The chance of completing the procedure within 100 min was not affected by time.

**Conclusion:**

For patients undergoing elective cholecystectomy after acute cholecystitis, the safety of the procedure increases if surgery is performed more than 30 days after discharge from the primary admission.

## Introduction

Although acute cholecystitis is a very common condition that is routinely managed at most acute surgery units, there are still controversies regarding the optimal management. Several randomized controlled trials state that acute surgery performed within 7 days may lead to shorter hospital stay when compared to elective surgery [1–3]. Similar conclusions were also drawn in a meta-analysis [4]. However, these studies have been based on patients in a stable general condition, fit for surgery, with low comorbidity and mild to moderate cholecystitis. Furthermore, the safety of delayed surgery can only be assessed if the timing of surgery is optimized. In one of the largest randomized controlled trials published so far, patients randomized to delayed surgery underwent planned cholecystectomy 7–45 days after the primary admission, which may be questioned as this is the period of maximum inflammatory response and the least suitable time for intervention [5]. Moreover, it has been reported that in the clinical setting, patients selected for early cholecystectomy tend to be younger, have lower comorbidity and tend to have lower grade of inflammation [6, 7].

Acute cholecystitis is a heterogeneous disease, ranging from mild inflammation in a stable patient to severe disease with septicemia, organ failure and locally advanced inflammation [8]. Some patients seek medical care with more than ten day’s symptom duration. In such cases, acute surgery may be complicated [9]. Many patients admitted with acute cholecystitis are not operated acutely due to severity of the cholecystitis, long history or lack of optimal resources at the time [6]. Even with ambition to perform acute surgery on patients clinically fit enough, there will inevitably be patients who benefit from conservative treatment in the acute setting followed by delayed elective surgery.

In most trials studying early versus delayed surgery, 6–12 weeks following primary admission have been chosen as the optimal time for delayed surgery, based on the belief that inflammation and fibrotic adhesions resolve in Calot´s triangle by that time [1, 2, 7]. To our knowledge, optimal timing for delayed cholecystectomy has only been investigated in one small study previously [10]. The aim of this study was to determine the optimal time to perform elective cholecystectomy after an episode of acute cholecystitis.

## Material and methods

**Fig. 1:**
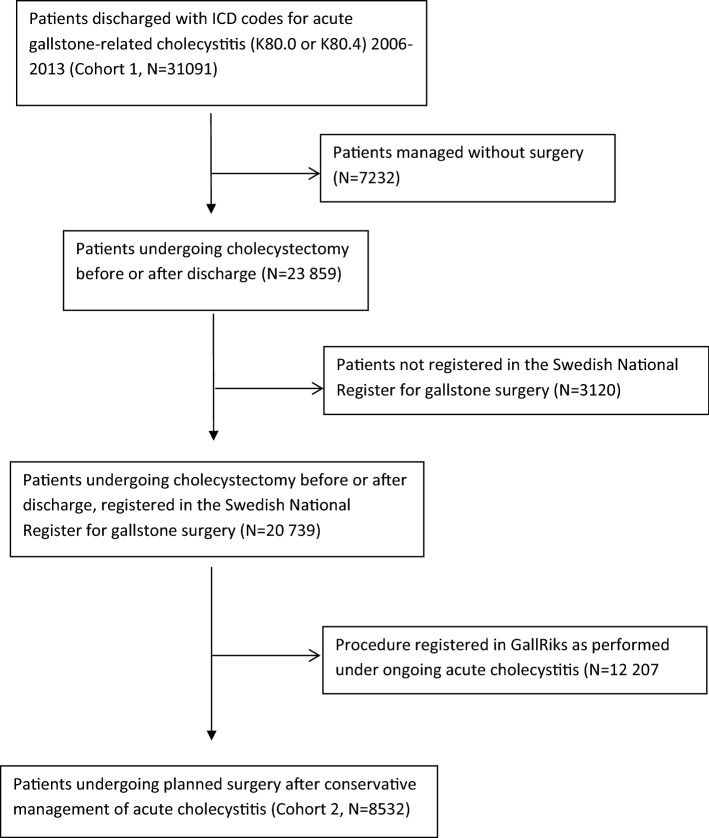
Flow chart of cohort assembly

All patients registered in the Swedish Patient Register (SPR) 2006–2013 with an ICD code indicating acute gallstone-related cholecystitis (K80.0 or K80.4) were included in the study. This cohort was cross-linked with the Swedish Register for Gallstone Surgery (GallRiks) using personal registration number and date of admission. A flow chart of cohort assembly is shown in Fig. 1.

GallRiks is a prospective database that includes patients undergoing cholecystectomy and ERCP in Sweden. GallRiks was started in 2005, and since 2009, it has become nationwide, covering more than 90% of all cholecystectomies performed in the country [11]. Registration of data is performed online by the surgeon immediately after surgery. There is also a mandatory 30-day follow-up by the local coordinator at each surgical unit participating in GallRiks. Validity of data in GallRiks is controlled on a regular basis and prospectively assessed by an independent audit group [11]. The NPR was started in the 1960´s aiming to cover all patients admitted to hospital, and registration became mandatory for all county authorities in Sweden in 1984. It has a coverage of more than 90% and high data validity. For surgical patients, indications for administration discharge, surgical procedures and ICD codes are collected at discharge and registered [12]. In this study, information on discharge from hospital after an episode of acute cholecystitis was retrieved from the NPR and the timing of surgery and outcome were retrieved from GallRiks.

Data on procedures completed with a minimally invasive technique, intra- and postoperative complications, time of surgery and bile duct injury and leakage were obtained from GallRiks. An intraoperative complication was defined as an adverse event registered during surgery, and a postoperative complication defined as a complication occurring within 30 days of surgery. Complications registered were haemorrhage, infection, bile duct injuries and leakage.

For the purpose of outcome comparisons, all data were divided into six-time interval categories between primary admission and elective surgery: 0–10; 11–30; 31–90; 90–180; 181–364; and > 365 days after primary admission; Day 0 being the day of discharge. The cut-off points were chosen as round-offs of equally distributed exponential intervals.

The study was reported according to the STROBE statement.

### Statistics

The impact of time interval between primary admission and elective surgery on operation time (longer or shorter than median), percentage of procedures completed as a minimally invasive technique (laparoscopic or minilaparotomy), intra- or postoperative complication and bile duct injury or bile leakage as outcome variables was assessed in univariate and multivariate logistic regression analyses. Each of the outcome variables was tested in separate analyses for each of the six-time interval categories. Multivariate logistic regression analyses of time to surgery were performed with the same outcome variables, adjusting for gender and age. Category 0–10 days was used as a reference category. Calculations were performed using SPSS Statistics 24.

## Results

Between 2006 and 2013, 31 091 patients were admitted for acute cholecystitis in Sweden (Cohort 1). Of these, 7232 were treated conservatively, 3120 patients were not registered in GallRiks, and 12,207 patients were treated with acute cholecystectomy. A total of 8532 patients were treated with elective surgery following an episode of acute cholecystitis (Cohort 2, Fig. 1). Patient characteristics for the elective surgery group are presented in Table 1. The majority of patients in this group were females. There were 196 (2.3%) cases of bile duct injury or bile leakage. 14.9% of patients had an intra- or postoperative complication. The distribution of patients for each time interval is presented in Table 2. Almost 50% of the patients were operated within 10 days after discharge (Table 2).

**Table 1:** Patient characteristics (Cohort 2, *N* = 8532)

Mean age, years (standard deviation)	54.1 (16.3)
*Gender*	
Men	3652 (42.8%)
Women	4880 (57.2%)
*Management*	
Laparoscopic	6564 (76.9%)
Laparoscopic, conversion to open	1120 (13.1%)
Minilaparotomy	102 (1.2%)
Open	716 (8.4%)
Other/information missing	20 (0.2%)
*Mean operating time,*	
Minutes (standard deviation)	113 (55)
Intra-/postoperative complication	1271 (14.9%)
Bile duct injury/bile leakage	196 (2.3%)

**Table 2:** Distribution of patients (Cohort 2) over time

Days	Frequency	Per cent	Cumulative per cent
0–10	4078	47.8	47.8
11–30	306	3.6	51.4
31–90	1693	19.8	71.2
91–180	1485	17.4	88.6
181–365	671	7.9	96.5
> 365	299	3.5	100
total	8532	100	

Surgical outcomes related to time interval category after discharge in the univariate and multivariate logistic regression analyses are presented in Tables 3, 4, 5, 6, 7. Multivariate analyses were performed with adjustment for age and gender. Altogether 17 patients died within 30 days after the cholecystectomy. Time from primary admittance to surgery did not have any statistically significant impact on postoperative mortality, neither in univariable not in multivariable logistic regression analysis (data not shown).

**Table 3:** Procedures completed as a minimally invasive technique

	Incidence	Univariate analysis		Multivariate analysis	
		Odds ratio (95% confidence interval)	*p*	Odds ratio (95% confidence interval)	*p*
*Time elapsed from discharge to surgery (days)*					
0–10 (Reference)	2964/4069 (72.8%)				
11–30	247/305 (81.0%)	1.588 (1.183–2.131)	0.002	1.637 (1.210–2.2124)	0.001
31–90	1416/1684 (84.1%)	1.970 (1.699–2.283)	< 0.001	2.277 (1.956–2.651)	< 0.001
91–180	1242/1478 (84.0%)	1.962 (1.680–2.292)	< 0.001	2.467 (2.102–2.897)	< 0.001
181–365	559/669 (83.6%)	1.895 (1.527–2.351)	< 0.001	2.363 (1.894–2.948)	< 0.001
> 365	238/297 (83.6%)	1.504 (1.122–2.016)	0.006	1.816 (1.344–2.454)	< 0.001
*Sex*					
Women (reference)	3967/4869 (81.5%)				
Men	2699/3633 (74.3%)	0.657 (0.592–0.729)	< 0.001	0.690 (0.619–0.768)	< 0.001
*Age*					
< Median (reference)	3661/4246 (86.2%)				
≥ Median	3005/4256 (70.6%)	0.384 (0.344–0.428)	< 0.001	0.350 (0.313–0.392)	< 0.001

**Table 4:** Procedures exceeding 100 min

	Incidence	Univariate analysis		Multivariate analysis	
		Odds ratio (95% confidence interval)	*p*	Odds ratio (95% confidence interval)	*p*
*Time elapsed from discharge to surgery (days)*					
0–10 (Reference)	2004/4078 (49.1%)				
11–30	148/306 (48.4%)	0.969 (0.768–1.223)	0.793	0.959 (0.759–1.211)	0.723
31–90	870/1693 (51.4%)	1.094 (0.977–1.225)	0.120	1.059 (0.945–1.187)	0.325
91–180	752/1485 (50.6%)	1.062 (0.943–1.196)	0.323	1.000 (0.887–1.128)	0.997
181–365	356/671 (53.1%)	1.170 (0.993–1.377)	0.060	1.108 (0.940–1.306)	0.223
> 365	151/298 (50.7%)	1.063 (0.840–1.345)	0.610	1.013 (0.800–1.283)	0.914
*Sex*					
Women (reference)	2313/4879 (47.4%)				
Men	1968/3652 (53.9%)	1.296 (1.190–1.413)	< 0.001	1.261 (1.157–1.376)	< 0.001
*Age*					
< median (reference)	1990/4255 (46.8%)				
≥ median	2291/4276 (53.6%)	1.314 (1.207–1.430)	< 0.001	1.275 (1.170–1.390)	< 0.001

**Table 5:** Perioperative complications

	Incidence	Univariate analysis		Multivariate analysis	
		Odds ratio (95% confidence interval)	*p*	Odds ratio (95% confidence interval)	*p*
*Time elapsed from discharge to surgery (days)*					
0–10 (Reference)	651/4078 (16%)				
11–30	41/306 (13.4%)	0.814 (0.580–1.144)	0.236	0.811 (0.576–1.141)	0.229
31–90	232/1693 (13.7%)	0.836 (0.711–0.983)	0.030	0.788 (0.670–0.928)	0.004
91–180	214/1485 (14.4%)	0.886 (0.750–1.048)	0.158	0.801 (0.676–0.949)	0.010
181–365	96/671 (14.3%)	0.879 (0.697–1.108)	0.275	0.797 (0.631–1.007)	0.057
> 365	37/299 (14.9%)	0.743 (0.522–1.059)	0.101	0.682 (0.478–0.974)	0.035
*Sex*					
Women (reference)	665/4880 (13.6%)				
Men	606/3652 (16.6%)	1.261 (1.119–1.421)	< 0.001	1.208 (1.071–1.364)	0.002
*Age*					
< median (reference)	495/4255 (11.6%)				
≥ median	776/4277 (18.1%)	1.684 (1.491–1.902)	< 0.001	1.699 (1.502–1.923)	< 0.001

**Table 6:** Postoperatively confirmed bile duct injuries

	Incidence	Univariate analysis		Multivariate analysis	
		Odds ratio (95% confidence interval)	*p*	Odds ratio (95% confidence interval)	*p*
*Time elapsed from discharge to surgery (days)*					
0–10 (Reference)	21/4078 (0.5%)				
11–30	2/306 (0.7%)	1.271 (0.297–5.446)	0.747	1.240 (0.289–5.316)	0.772
31–90	9/1693 (0.5%)	1.032 (0.472–2.259)	0.936	0.979 (0.452–2.169)	0.990
91–180	13/1485 (0.9%)	1.706 (0.852–3.416)	0.131	1.572 (0.781–3.166)	0.205
181–365	4/671 (0.6%)	1.159 (0.396–3.386)	0.788	1.079 (0.368–3.165)	0.889
> 365	5/299 (1.7%)	3.286 (1.230–8.776)	0.018	3.075 (1.147–8.241)	0.026
*Sex*					
Women (reference)	24/4880 (0.5%)				
Men	30/3652 (0.8%)	1.676 (0.978–2.871)	0.060	1.582 (0.919–2.721)	0.098
*Age*					
< median (reference)	22/4255 (0.5%)				
≥ median	32/4277 (0.7%)	1.450 (0.841–2.500)	0.181	1.314 (0.756–2.283)	0.333

**Table 7:** Postoperatively confirmed cystic duct leakage

	Incidence	Univariate analysis		Multivariate analysis	
		Odds ratio (95% confidence interval)	*p*	Odds ratio (95% confidence interval)	*p*
*Time elapsed from discharge to surgery (days)*					
0–10 (Reference)	40/4078 (1.0%)				
11–30	4/306 (1.3%)	1.337 (0.475–3.762)	0.582	1.302 (0.462–3.667)	0.617
31–90	5/1693 (0.3%)	0.299 (0.118–0.759)	0.011	0.295 (0.116–0.750)	0.010
91–180	7/1485 (0.5%)	0.478 (0.214–1.070)	0.072	0.465 (0.207–1.045)	0.064
181–365	3/671 (0.4%)	0.453 (0.140–1.470)	0.187	0.445 (0.137–1.448)	0.179
> 365	2/299 (0.7%)	0.680 (0.163–2.827)	0.596	0.668 (0.160–2.776)	0.577
*Sex*					
Women (reference)	30/4880 (0.6%)				
Men	31/3652 (0.8%)	1.384 (0.836–0.291)	0.206	1.457 (0.875–2.426)	0.148
*Age*					
< median (reference)	32/4255 (0.8%)				
≥ median	29/4277 (0.7%)	0.901 (0.544–1.492)	0.685	0.955 (0.572–1.595)	0.955

**Fig. 2:**
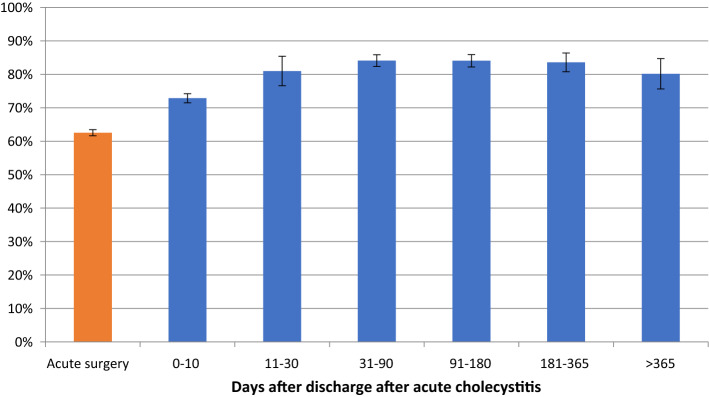
Procedures completed as a minimally invasive technique. Error bars 95% CI

**Fig. 3:**
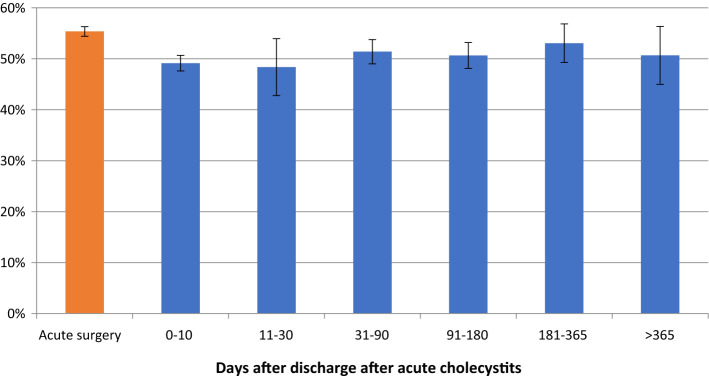
Procedures exceeding 100 min. Error bars 95% CI

**Fig. 4:**
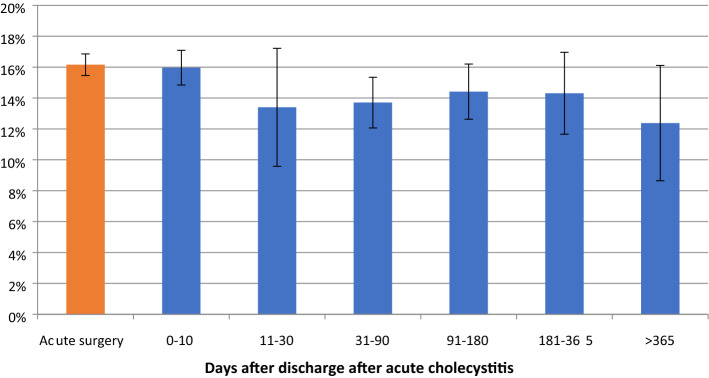
Perioperative complications. Error bars 95% CI

**Fig. 5:**
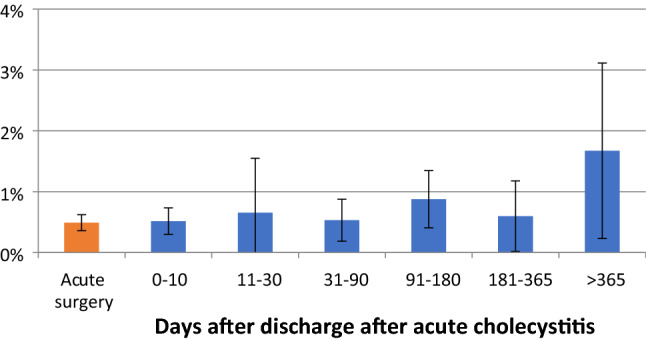
Bile duct injury. Error bars 95% CI

**Fig. 6:**
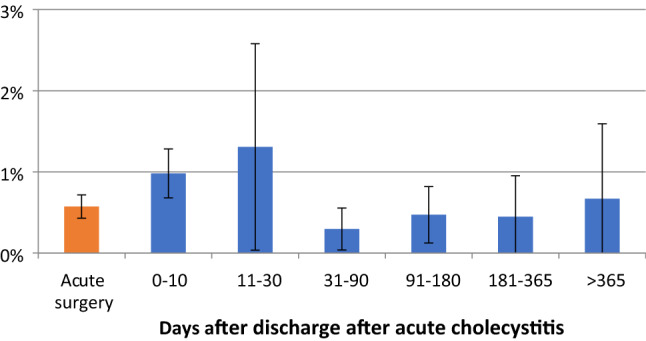
Cystic leakage. Error bars 95% CI

**Fig. 7:**
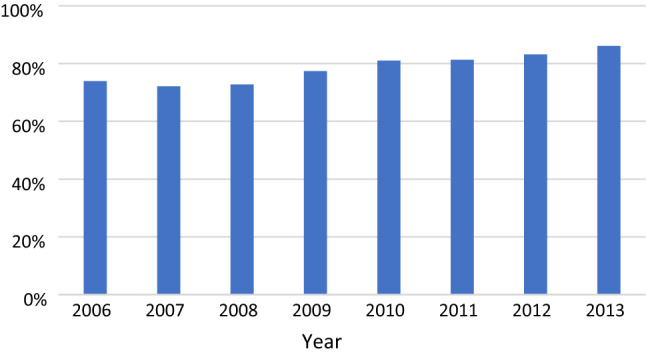
Surgeries completed laparoscopically over the study period

For procedures performed after 30 days, there was a 50% reduction in the number of procedures not completed as a minimally invasive technique (Fig. 2, Table 3). The percentage of procedures exceeding 100 min was relatively stable throughout the study period (Fig. 3, Table 4). Perioperative complication rates showed a tendency to decrease with time, becoming statistically significant after 30 days in the multivariate analysis (Fig. 4, Table 5). Bile duct injury rates fell steadily up to one year, but the only statistically significant decrease was seen in the > 365 day category. Variability was great in the group operated more than one year after discharge (Fig. 5, Table 6). After 30 days, there was a decrease in cystic duct leakage (Fig. 6, Table 7). During the study period, the number of procedures completed laparoscopically increased (Fig. 7.)

## Discussion

This study on the optimal timing of elective cholecystectomy after conservatively treated acute cholecystitis showed that the risk for intra- and postoperative complication, bile duct injury, bile leakage and number of procedures not completed as a minimally invasive technique decreases with time after primary admission. Based on these observations, elective surgery should preferably be performed at least 30 days after discharge from the primary admission.

Although widely accepted guidelines recommend immediate surgery for acute cholecystitis, the outcome of delayed cholecystectomy must be considered as this is still common clinical practice. As acute cholecystitis is a heterogeneous disease affecting not only young patients who are fit for immediate surgery but also older patients with high-risk comorbidity conservative management will often be considered. Delayed surgery is also recommended for those presenting with a long history of symptoms [13]. Most studies comparing early and delayed cholecystectomy have recommended a fixed point in time for delayed cholecystectomy, but do not answer the question what is optimal timing for delayed cholecystectomy after acute cholecystitis [2, 5].

Despite recommendations in favour of early cholecystectomy, 45–67% of all patients presenting with acute cholecystitis are treated conservatively [6, 7, 14]. The reasons for this vary, from those related to the condition of the individual patient to those related to the healthcare system in general. Whereas most of the randomized studies published so far suggest that early cholecystectomy is a safe procedure [4]. The favourable outcome has not been reproduced in population-based studies [6, 15, 16]. The discrepancy between the randomized controlled trials and the population-based studies may be explained by the higher external validity of the latter ones as they reflect the outcome of surgery in the community at large, not only what is achieved at highly specialized centres with narrow patient selection and surgeons and crew devoted to the care of patients with AC.

There are few studies addressing this matter. It has been shown that increased severity of acute cholecystitis is associated with an increased risk for bile duct injury. Compared to mild inflammation where no increase in risk for bile duct injuries was noted while performing acute surgery, the risk doubled in patients presenting with moderate inflammation and was even higher in those with severe inflammation [17]. The actual benefit of acute surgery in patients 75 years and older is also unclear. Compared to younger patients, this group suffers higher morbidity and mortality when acute cholecystectomy is performed. Careful consideration of the benefits and dangers should therefore be taken before acute surgery is attempted in these patients [18–21].

To our knowledge, this is the largest study exploring this issue. Only one previous study performed by Hershkovitz et al. addressed this question. They studied 213 patients retrospectively. The patients were divided into three-time interval categories: 1–6 weeks; 6–12 weeks; and more than 12 weeks. They did not find any statically significant difference between these groups but a trend towards safer surgery in the early and late categories [10]. In our study, we found a statistically significant increase in procedures completed as a minimally invasive technique as time proceeded. After 30 days, a decrease in intra- and postoperative complication rates was seen, and in the 31–90 days category, a significant decrease in bile leakage rates was also seen. We did not find any significant difference in operating time between the six categories. From these results, we draw the conclusion that elective cholecystectomy should be performed at least 30 days after admission for acute cholecystitis.

In the present study, we did not take recurrent cholecystitis into account, as the study period covered the time period from the last episode of cholecystitis to definitive surgery. In order to avoid recurrent cholecystitis, the risk for a new episode of cholecystitis has to be taken into consideration when choosing the timing of elective surgery. We cannot predict the risk of recurrent cholecystitis since many of the patient underwent planned surgery before the recurrence or were not followed long enough to develop recurrence. In a previous study, we found that the rate of recurrence of cholecystitis was approximately 18% within one year, with most recurrences occurring within 6 months [22]. This is in agreement with the results of several other studies [23]. Elective surgery should therefore be scheduled without unnecessary delay in order to avoid a new episode of acute cholecystitis.

We found in this study fewer serious complications in the delayed group than in the group operated on acutely, which contradicts the findings of previous studies where a lower risk for complications has been reported with acute surgery [2, 5, 7]. However, no definite conclusion can be drawn from this as we do not have information on the selection process in this study. We suspect selection bias which would be a great limitation should that have been the case. In a recent systemic review of meta-analyses, no significant difference was found in overall complication rates between acute and delayed surgery [24]. In one of the largest randomized controlled studies on acute vs delayed cholecystectomy (the ACDC study), morbidity rates were significantly lower in the acute surgery group (within 24 h of admission) than in the delayed group [5]. However, the patients in the delayed group were scheduled for surgery days 7–45 which does not concur with the period previously considered to be safe, i.e. when inflammation has subsided. In our study, patients operated within 10 days had the highest risk for complications; the risk subsided after 30 days. We also observed a high percentage of cases in the acute surgery group where surgery was not completed as a minimally invasive technique, higher than reported in several previous studies [5, 15]. Since our study included both small and large Swedish hospitals, we believe our results reflect reality in day-to-day clinical practice outside specialized centres. The relatively high conversion rate may, to some extent, be explained by the lack of routine to perform laparoscopic cholecystectomy for acute cholecystitis during the first year of the study period. We noticed a reduction in perioperative complications during the study period and see this as a measure for difficult dissection. We found that 17 patients died within 30 days after the cholecystectomy. We do not have information on the cause of death for these patients, but in a previous study, 9.7% of patients dying within 30 days after surgery were assumed to have done so due to postoperative complications [25].

We found that a large proportion of patients were operated on within 10 days after discharge for acute cholecystitis. These patients were recorded as discharged and subsequently registered in GallRiks as elective surgery, but we cannot exclude the possibility that some of these were patients undergoing acute surgery during hospitalization for acute cholecystitis. It is also possible that some of them were transferred between two units and therefore registered as two admissions. It has been described in previous studies that conservative treatment with antibiotics for acute cholecystitis has a failure of approximately 20%. This could explain the high rate of patients undergoing surgery within 10 days in the present study [26].

The study has some limitations and confounders that must be taken into account. Although GallRiks is continuously validated and accuracy has been found to be high for all variables, the inclusion criteria for the present study were based on a subgroup in GallRiks where selection may be affected by bias not covered by validation routines. GallRiks does not include information on the histology, BMI or classification of cholecystitis according to Tokyo guidelines which may have served as confounders. This could have caused a bias since the decision to postpone surgery may have been related to the general condition of the patient.

The outcomes of the study are largely dependent on early readmission after a primary admission when the decision was taken not to perform acute cholecystectomy. There may have been a number of factors influencing the primary decision to refrain from surgery, this decision being reconsidered when the patient was readmitted. There was a relatively large number of patients that were readmitted shortly after primary admission, this group had a relatively large impact on the outcomes in this study. We also do not have information on comorbidity, the severity of inflammation and when patients present with symptoms.

The cholecystitis diagnosis was in most cases determined by macroscopic examination. The specimens are routinely sent for histopathologic examination in 44% of the cases [27]. Although histopathologic examination is necessary to obtain definite diagnosis, the decision to carry out surgery was based on clinical assumption of cholecystitis. We do not have data on incidental finding of malignancies in the present cohort. The specimens were sent for histopathological examination selectively, which is the usual routine in Sweden. With this approach, the prevalence of incidentally detected gallbladder carcinoma was found to be 0,26% of the specimens analysed. [27]

In the small category operated on more than one year after admission, we saw an increased risk for bile duct injury and bile leakage, but the variation was large, shown by the wide confidence interval. As we do not have information on why it was decided to perform surgery such a long time after admission for acute cholecystitis, there may have been special circumstances that lay behind this decision, resulting in strong selection bias.

The data in this study are until year 2013. Surgical safety has probably advanced since then. The laparoscopic skills of surgeons performing cholecystectomies have improved and the equipment used for surgery developed. Nevertheless, the inflammatory changes occurring during the first weeks after an acute cholecystitis remain the same thus making surgery still safer if performed after 30 days. The same benefit of postponing surgery is thus probably valid also for laparoscopic surgery as it is carried out today.
